# Real-world analysis of gender differences in drug-induced insomnia: evidence from FAERS and CVARDD databases

**DOI:** 10.3389/fneur.2025.1702264

**Published:** 2026-02-02

**Authors:** Yuntai Wang, Shengjie Wang, Fuxing Liu

**Affiliations:** 1 Department of Rehabilitation Medicine, Hubei Provincial Hospital of Integrated Chinese and Western Medicine, Wuhan City, Hubei Province, China; 2 Department of Rehabilitation Medicine, Xinhua Hospital of Hubei University of Chinese Medicine, Wuhan City, Hubei Province, China

**Keywords:** disproportionality analysis, drug-induced insomnia, FAERS database, gender differences, medication safety, real-world data

## Abstract

**Background:**

Insomnia is a common sleep disorder that substantially impairs quality of life. Drug-induced insomnia (DII), an important cause of secondary insomnia, is often underrecognized, and many potential signals are not yet documented in drug labels. Evidence regarding sex-specific differences in DII remains limited, hindering the development of tailored safety strategies.

**Objective:**

To identify drug–insomnia associations, assess sex-specific differences, validate signals in an independent database, and characterize the time-to-onset (TTO) of high-risk drugs using large-scale real-world pharmacovigilance data.

**Methods:**

We conducted a retrospective observational pharmacovigilance study using insomnia-related reports from FAERS (2004Q1–2025Q2). Disproportionality analyses (ROR, PRR, BCPNN, MGPS) were performed, and sex-stratified associations were compared using Wald chi-square tests. Signals were externally validated in the Canadian Vigilance Adverse Reaction Database (CVARDD). Weibull models were applied to evaluate TTO for the drugs with the highest insomnia report counts.

**Results:**

A total of 266,429 insomnia-related reports were identified, with more reports from females (60.1%) than males (32.0%). A total of 237 drugs demonstrated significant disproportionality signals, including several without labeled insomnia risk. Among the 20 most frequently implicated drugs, 15 showed significant sex–drug interactions. Duloxetine exhibited a stronger association in males, whereas niraparib and levothyroxine showed higher risks in females. External validation confirmed 124 overlapping drugs with consistent signals. TTO analyses revealed an early-failure pattern (Weibull β < 1) for all five high-reporting drugs, with median onset ranging from 3 to 211.5 days.

**Conclusion:**

This study identified multiple drug–insomnia signals, quantified sex-specific differences, and validated findings in an independent database. These results underscore the importance of recognizing DII and monitoring sex-related variability in clinical practice.

## 1 Introduction

Insomnia is one of the most prevalent sleep disorders worldwide, characterized by difficulties initiating or maintaining sleep or experiencing early morning awakenings with impaired functional recovery. It is associated with daytime fatigue, cognitive dysfunction, mood disturbances, and reduced overall quality of life (1, 2). Global prevalence estimates from the World Health Organization indicate that 10–30% of adults experience insomnia symptoms, with the burden continually increasing due to population aging, lifestyle acceleration, and rising psychological stress (3, 4).

Among the diverse causes of insomnia, drug-induced insomnia (DII)—sleep disturbances attributable to medication use—represents a clinically important yet frequently underrecognized contributor to secondary insomnia (5). The onset of DII is closely linked to pharmacological mechanisms, dosing patterns, and individual variability (6). For instance, glucocorticoids may disrupt sleep by altering serotonin release in the dorsal raphe nucleus and interfering with suprachiasmatic nucleus (SCN) regulation, while the insomnia-related effects of statins vary according to blood–brain barrier penetration and metabolic alterations (7, 8). Although some medications include insomnia in their product labeling, many additional agents may elicit sleep disturbances but remain undocumented in regulatory frameworks or clinical practice.

A growing body of evidence highlights sex differences as critical determinants of medication response and safety. Women and men differ in sex hormone profiles, neurotransmitter regulation, immune responses, and cytochrome P450 enzyme expression, all of which influence drug pharmacokinetics and pharmacodynamics (9–11). Sleep physiology also shows substantial sex specificity, with women demonstrating greater vulnerability to sleep disturbances during menstrual cycles, pregnancy, and menopause transitions (12, 13). Recent epidemiological and sleep research has demonstrated pronounced sex differences in insomnia, with women exhibiting a higher prevalence, greater vulnerability across developmental stages, and distinct physiological and circadian features compared with men (14, 15). Despite such well-established sex-related differences in sleep regulation, very few studies have systematically examined drug-induced insomnia using sex-stratified analyses. Existing evidence remains fragmented and largely descriptive, as previous reviews have noted that research on medication-related sleep disturbances has predominantly focused on individual drug classes rather than applying systematic, population-level approaches (5). This gap highlights the need for structured, large-scale investigations into sex-specific patterns of DII.

Real-world data (RWD), particularly from large spontaneous reporting systems, offer valuable opportunities to detect potential adverse drug reaction signals at the population level. The FDA Adverse Event Reporting System (FAERS) is one of the largest global pharmacovigilance databases and has been widely used to detect safety signals across diverse therapeutic areas. Similarly, the Canadian Vigilance Adverse Reaction Database (CVARDD) provides independent national-level data for validating signal robustness (16).

Accordingly, the present study aimed to identify drug–insomnia associations, assess sex-specific differences, validate the detected signals in an independent database, and characterize the time-to-onset of high-risk drugs using large-scale real-world pharmacovigilance data.

## 2 Materials and methods

### 2.1 Data source and process

This is a retrospective observational study based on spontaneous reports from the FDA Adverse Event Reporting System (FAERS)(https://fis.fda.gov/extensions/FPD-QDE-FAERS/FPD-QDE-FAERS.html). Insomnia-related adverse event reports from 2004Q1 to 2025Q2 were extracted and analyzed. To assess the reproducibility of detected signals, we conducted external validation using the Canadian Vigilance Adverse Reaction Database (CVARDD). CVARDD was selected because it is a national spontaneous reporting system with structural and operational similarities to FAERS, yet represents a distinct regulatory environment and patient population. Consistency between FAERS- and CVARDD-derived signals strengthens the robustness and generalizability of the findings. This is a retrospective observational study based on spontaneous reports from the FDA Adverse Event Reporting System (FAERS).

For records sharing the same caseid, only the most recent report was retained following standard FAERS deduplication procedures. Drug names were standardized using the RxNorm terminology system to merge spelling variations, brand names, and generic names. Adverse events were coded using MedDRA version 27.1, and insomnia cases were identified using a predefined set of Preferred Terms (PTs), including:

“Insomnia,” “Middle insomnia,” “Terminal insomnia,” “Initial insomnia / Sleep initiation disorder,” “Early morning awakening,” “Psychophysiologic insomnia,” “Paradoxical insomnia,” “Behavioural insomnia of childhood,” “Insomnia related to another mental condition,” “Sleep disorder due to general medical condition, insomnia type,” “Fatal familial insomnia.”

Reports containing any of these PTs were classified as insomnia. When multiple adverse events were reported, the presence of any insomnia-related PT qualified the report as an insomnia case.

To reduce attribution bias, only Primary Suspect (PS) drugs were included for signal detection. Reports with multiple PS drugs were counted once for each PS drug. All standardized insomnia reports were further characterized by sex, age, weight, indication, reporting country, and clinical outcomes.

### 2.2 Signal analysis algorithms

This study employed disproportionality analysis (DPA), a widely used pharmacovigilance data-mining approach, to identify potential signals of insomnia-related adverse drug events (ADEs). DPA evaluates whether the reporting frequency of a drug–event combination exceeds the expected background frequency using 2 × 2 contingency tables (see Supplementary material 1). Four commonly used algorithms were applied (13): Reporting Odds Ratio (ROR), Proportional Reporting Ratio (PRR), Bayesian Confidence Propagation Neural Network (BCPNN), Multi-item Gamma-Poisson Shrinkage (MGPS), ROR is advantageous in handling sparse data and can partially mitigate biases due to low reporting counts (17). PRR provides higher specificity in detecting disproportionality (18). BCPNN uses Bayesian inference to estimate information components (IC) with stable statistical properties (19). MGPS is suitable for signal detection involving rare or low-frequency events.

All formulas, shrinkage procedures, and statistical thresholds are detailed in Supplementary Table 1. To avoid zero-cell bias, a Haldane–Anscombe correction (adding 0.5 to each cell when a zero value was present) was applied. A DPA signal was considered present when: the lower bound of the 95% CI of ROR was >1, and the frequency of the target cell (a) was >3 (20).

To reduce false positives due to multiple testing, *p*-values were adjusted using the Benjamini–Hochberg false discovery rate (FDR) method via the “p.adjust” function in the R stats package, with statistical significance defined as FDR-adjusted *p* < 0.05 (21).

Detected signals were compared against official FDA labeling information (https://www.accessdata.fda.gov/scripts/cder/daf/index.cfm). Signals not listed on the label were classified as potential new insomnia-related adverse reactions. All analyses were conducted using R version 4.4.0 and Microsoft Excel.

### 2.3 Sex-specific analyses and interaction testing

To formally evaluate sex differences—beyond descriptive stratification—we conducted sex-specific disproportionality analyses and statistical tests for sex–drug interactions. For each drug, separate 2 × 2 contingency tables were generated for males and females, defined as follows:

a = number of insomnia reports associated with the drug.

b = number of non-insomnia reports associated with the drug.

c = number of insomnia reports associated with all other drugs.

d = number of non-insomnia reports associated with all other drugs.

Sex-specific RORs were calculated as:


$$ROR=\frac{a\times d}{b\times c}$$

The variance of the log-transformed ROR was estimated using:


$$\mathrm{Var}(\ln\mathrm{ROR})=\frac{1}{a}+\frac{1}{b}+\frac{1}{c}+\frac{1}{d}$$

To identify whether the drug–insomnia association differed significantly between sexes, we compared the male and female log RORs using a Wald chi-square test for equality of sex-specific effects:

$$z=\frac{\ln\mathrm{RO}R_{m\mathrm{ale}}-\ln\mathrm{RO}R_{f\text{emale}}}{\sqrt{\mathrm{Va}r_{m\mathrm{ale}}+\mathrm{Va}r_{f\text{emale}}}}$$

$$\chi^{2}=z^{2},\mathrm{df}=1$$

**Figure 1:**
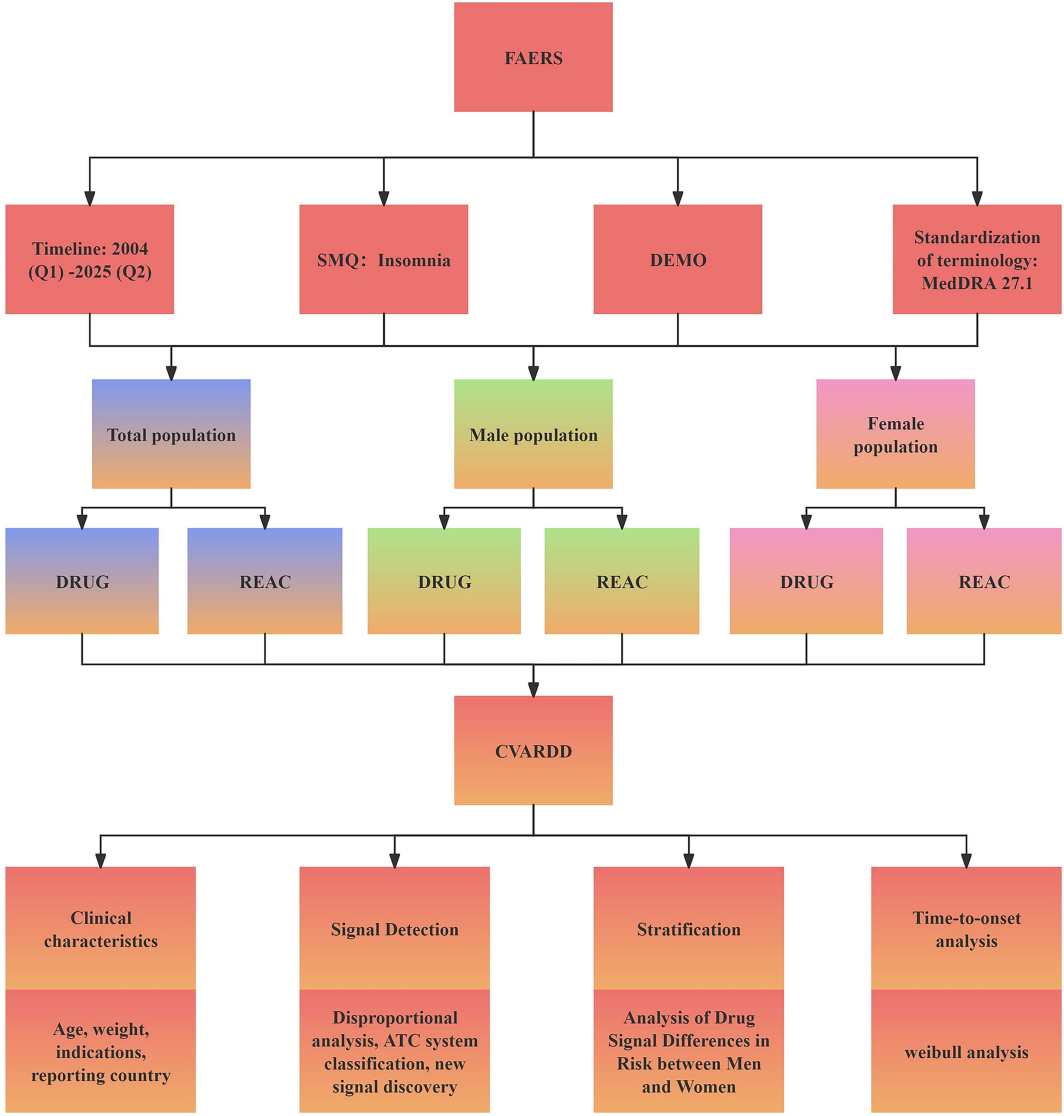
Process for disproportionate analysis of adverse reactions related to insomnia caused by drug-related factors. FAERS: FDA Adverse Event Reporting System, a spontaneous reporting database used for pharmacovigilance. SMQ: Standardized MedDRA Query, a predefined group of MedDRA terms used to identify adverse event categories—in this study, the EP-related SMQ. DEMO: Demographic Data Module in FAERS, containing patient-level variables such as age, sex, and reporting year. ATC: Anatomical Therapeutic Chemical Classification System, used for categorizing drugs by therapeutic class and mechanism.

A *p*-value < 0.05 indicated a significant sex–drug interaction, reflecting a statistically meaningful difference between male and female reporting associations. The data extraction and analysis workflow is shown in Figure 1.

## 3 Results

### 3.1 Basic characteristics of DII adverse events

**Figure 2:**
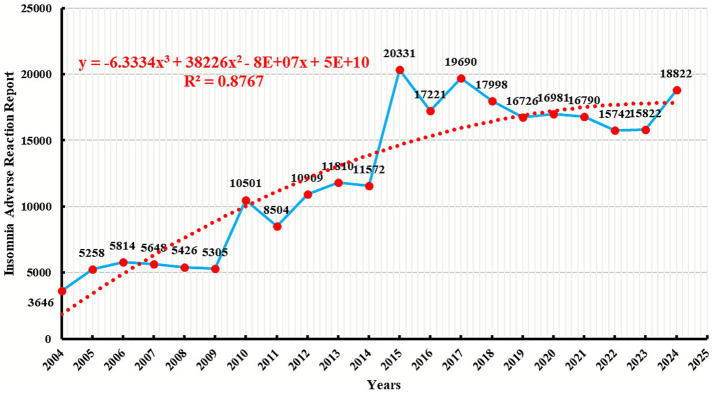
Trend of adverse event reports for drug related insomnia. The red dashed line represents the polynomial fitting curve; The blue solid line represents the number of reported adverse events.

As shown in Figure 2, since 2004, the overall number of reports of insomnia-related adverse events has shown an upward trend. In 2004, there were only 3,646 reports. This number fluctuated and gradually increased year by year, peaking in 2015 with 20,331 reports, and closely approaching this peak again in 2017 with 19,690 reports. Although there was a slight decrease afterward, the overall number remained at a high level, with 18,822 reports in 2024, which is significantly higher than in earlier years. To further predict the trend of future event reporting, a polynomial fitting curve was plotted. The polynomial curve indicates that the number of DII reports continues to rise. The coefficient of determination (R^2^ = 0.8767) means that the model can explain 87.67% of the variability in the data, making this trend of high reference value.

Table 1 shows the demographic characteristics of the population associated with DII adverse events. Among the 266,429 reports, 160,115 (60.1%) were from females, 85,282 (32.0%) were from males, and 21,032 (7.9%) were of unknown gender. In terms of age stratification, for reports with known age, the highest proportion (42.84%) was in the 18–65 years age group. The majority of DII adverse events were concentrated in the weight group ≥75 kg (19.96%), but 61.25% of the reports did not include weight information. Notably, the most frequently reported indication was “PRODUCT USED FOR UNKNOWN INDICATION” (13.01%), followed by “MULTIPLE SCLEROSIS” (3.40%) and “RHEUMATOID ARTHRITIS” (3.36%). The majority of DII adverse event reports came from developed countries, with the United States accounting for 70.88%, Canada for 7.19%, and Great Britain for 4.78%.

**Table 1:** Baseline characteristics of drug-related insomnia population.

Characteristics	Case numbers	Case proportion (%)
Number of events	*N* = 266,429	-
Gender	-	-
Female	160,115	60.1%
Male	85,282	32.0%
Miss	21,032	7.9%
Age	-	-
Median Age	55	
<18	7,266	2.73%
18–65	114,146	42.84%
65–85	46,375	17.41%
>85	4,286	1.61%
Miss	94,356	35.42%
Weight(KG)	-	-
<40	2,798	1.05%
40–65	28,004	10.51%
65–75	19,259	7.23%
≥75	53,187	19.96%
Miss	163,181	61.25%
Top 5 indication	-	-
product used for unknown indication	34,653	13.01%
Miss	27,702	10.40%
Multiple sclerosis	9,048	3.40%
Rheumatoid arthritis	8,940	3.36%
Depression	8,006	3.00%
Top 5 Reported Countries	-	-
United States	188,834	70.88%
Canada	19,155	7.19%
Great Britain	12,740	4.78%
France	6,021	2.26%
Brazil	3,486	1.31%

### 3.2 Analysis of insomnia risk drugs in the general population

**Figure 3:**
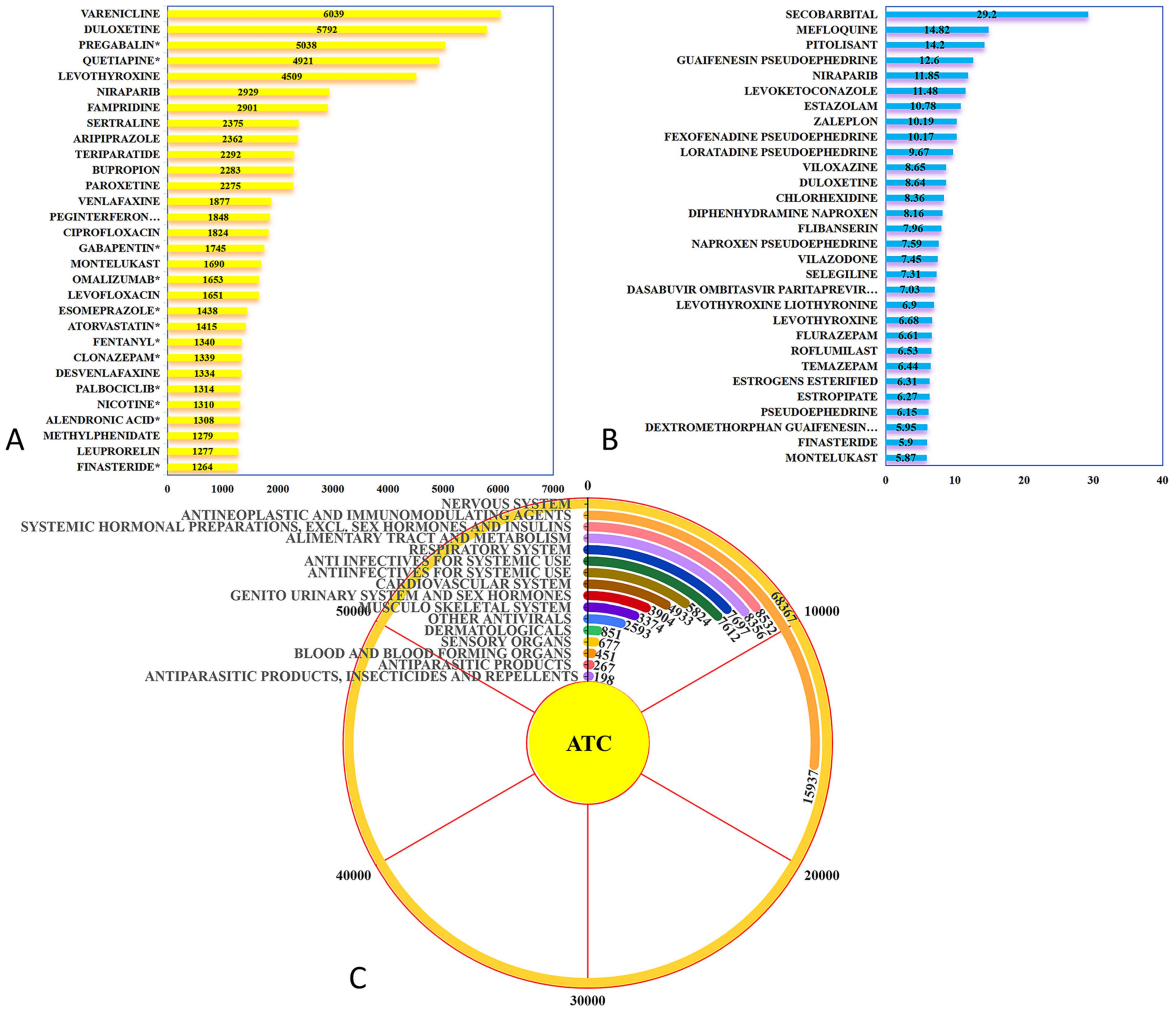
Insomnia risk drugs in the general population. **(A)** Top 20 drugs by number of reports for insomnia risk; **(B)** Top 20 drugs by ROR for insomnia risk; **(C)** ATC system classification of insomnia risk drugs in a pie chart.

There are 237 drugs associated with DII adverse events, as shown in Figure 3A. The top five drugs are METHOTREXATE (*N* = 578), DUPILUMAB (*N* = 547), CIPROFLOXACIN (*N* = 302), TERIPARATIDE (*N* = 285), and LEVOFLOXACIN (*N* = 236). As shown in Figure 3B, the drugs with the highest Reporting Odds Ratios (ROR) are SECOBARBITAL (ROR = 29.2), MEFLOQUINE (ROR = 14.82), PITOLISANT (ROR = 14.2), GUAIFENESIN PSEUDOEPHEDRINE (ROR = 12.6), and NIRAPARIB (ROR = 11.85). A classification of all risk drugs according to the ATC system is shown in Figure 3C. A total of 16 different ATC categories were identified, with the Nervous System category (*N* = 168,367) being the most frequent, followed by Antineoplastic and Immunomodulating Agents (*N* = 15,937). Further comparison with drug labels revealed that 11 drugs (marked with an asterisk in Figure 3A) such as PREGABALIN* (*N* = 5,038, ROR = 3.22), QUETIAPINE* (*N* = 4,921, ROR = 4.76), GABAPENTIN* (*N* = 1,745, ROR = 1.93), OMALIZUMAB* (*N* = 1,653, ROR = 2.01), and ESOMEPRAZOLE* (*N* = 1,438, ROR = 1.47) were not directly mentioned in their drug labels as being associated with insomnia-related adverse events. These new signals warrant further clinical attention.

### 3.3 Analysis of gender differences in risk drugs

**Figure 4:**
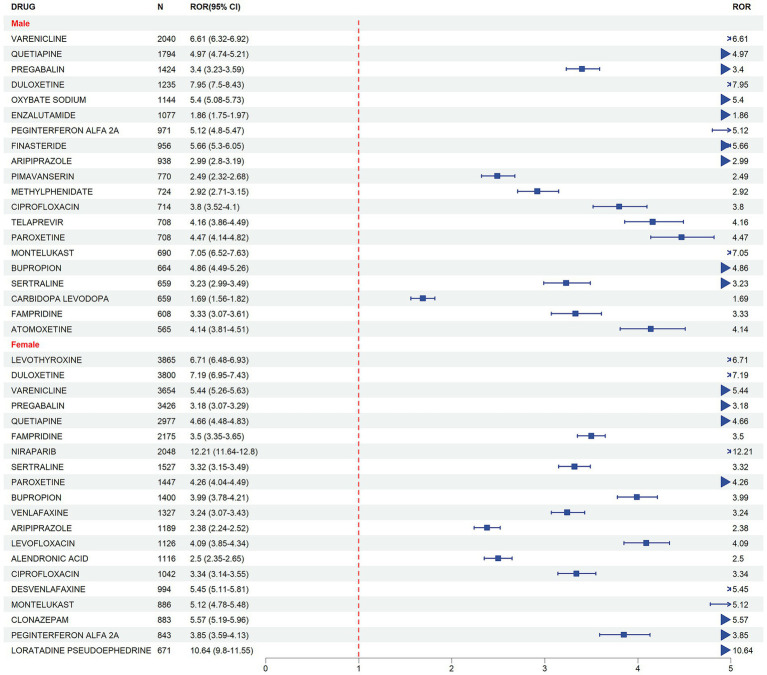
Forest of risk signals for insomnia in males and females.

To further clarify gender differences in DII risk signals, we organized the risk drugs for males and females. As shown in Figure 4, among male patients, DULOXETINE exhibited the highest insomnia risk (ROR = 7.95, 95% CI: 7.50–8.43), significantly higher than other drugs. In addition, MONTELUKAST (ROR = 7.05, 95% CI: 6.52–7.63), VARENICLINE (ROR = 6.61, 95% CI: 6.32–6.92), and PEGINTERFERON ALFA 2A (ROR = 5.12, 95% CI: 4.80–5.47) also showed high insomnia risk. Commonly used psychiatric drugs such as QUETIAPINE (ROR = 4.97), PAROXETINE (ROR = 4.47), and BUPROPION (ROR = 4.86) were significantly associated with insomnia, while ENZALUTAMIDE (ROR = 1.86) and CARBIDOPA LEVODOPA (ROR = 1.69) showed lower risk, albeit with some indication of potential risk.

As shown in Figure 4, in females, NIRAPARIB (ROR = 12.21, 95% CI: 11.64–12.80) presented a significantly higher risk, with no similar result observed in the male group. DULOXETINE (ROR = 7.19, 95% CI: 6.95–7.43) and LEVOTHYROXINE (ROR = 6.71, 95% CI: 6.48–6.93) also exhibited a strong insomnia risk. Additionally, DESVENLAFAXINE (ROR = 5.45, 95% CI: 5.11–5.81) and VARENICLINE (ROR = 5.44, 95% CI: 5.26–5.63) were highly correlated with insomnia in females. Similar to the male results, psychiatric drugs such as QUETIAPINE, PAROXETINE, BUPROPION, and SERTRALINE were also significantly related to insomnia in females.

To further reduce the risk of false positives, we further screened the top 30 risk drugs that were positive in all four algorithms: Reporting Odds Ratio (ROR), Proportional Reporting Ratio (PRR), Bayesian Confidence Propagation Neural Network (BCPNN), and Multi-item Gamma-Poisson Shrinkage (MGPS), as shown in Table 2. In males, the following drugs showed significant insomnia risk signals, with significant results even after multiple correction tests (*p* < 0.001): VARENICLINE (ROR = 6.61, 95% CI: 6.32–6.92), QUETIAPINE (ROR = 4.97, 95% CI: 4.74–5.21), PREGABALIN (ROR = 3.40, 95% CI: 3.23–3.59), DULOXETINE (ROR = 7.95, 95% CI: 7.50–8.43), and SODIUM OXYBUTYRATE (ROR = 5.40, 95% CI: 5.08–5.73). Similar risk patterns were observed in females, with QUETIAPINE, DULOXETINE, and PREGABALIN also appearing among the high-risk drugs, but with generally higher effect sizes in females. Notably, differences in the risk ranking of some drugs between genders suggest that males and females may have different susceptibilities to DII.

**Table 2:** Top 30 insomnia risk medications for men and women.

Gender	Drug	Case Count (n)	ROR (95% CI)	PRR (χ^2^)	EBGM (EB05)	IC (IC025)	*p*-value	Adjusted p-value
Male	VARENICLINE	2040	6.61 (6.32–6.92)	6.17 (8748.13)	6.05 (5.78)	2.6 (2.53)	<0.001	<0.001
	QUETIAPINE	1794	4.97 (4.74–5.21)	4.73 (5234.9)	4.65 (4.43)	2.22 (2.14)	<0.001	<0.001
	PREGABALIN	1,424	3.4 (3.23–3.59)	3.3 (2277.57)	3.26 (3.09)	1.71 (1.63)	<0.001	<0.001
	DULOXETINE	1,235	7.95 (7.5–8.43)	7.3 (6708.96)	7.21 (6.8)	2.85 (2.76)	<0.001	<0.001
	OXYBATE SODIUM	1,144	5.4 (5.08–5.73)	5.11 (3781.59)	5.06 (4.76)	2.34 (2.24)	<0.001	<0.001
	PEGINTERFERON ALFA 2A	971	5.12 (4.8–5.47)	4.86 (2985.03)	4.82 (4.52)	2.27 (2.17)	<0.001	<0.001
	FINASTERIDE	956	5.66 (5.3–6.05)	5.34 (3378.44)	5.29 (4.95)	2.4 (2.3)	<0.001	<0.001
	ARIPIPRAZOLE	938	2.99 (2.8–3.19)	2.91 (1180.65)	2.89 (2.71)	1.53 (1.43)	<0.001	<0.001
	PIMAVANSERIN	770	2.49 (2.32–2.68)	2.45 (660.69)	2.43 (2.26)	1.28 (1.17)	<0.001	<0.001
	METHYLPHENIDATE	724	2.92 (2.71–3.15)	2.85 (874.32)	2.84 (2.63)	1.5 (1.39)	<0.001	<0.001
	CIPROFLOXACIN	714	3.8 (3.52–4.1)	3.67 (1392.57)	3.65 (3.38)	1.87 (1.75)	<0.001	<0.001
	TELAPREVIR	708	4.16 (3.86–4.49)	4 (1600.27)	3.97 (3.68)	1.99 (1.87)	<0.001	<0.001
	PAROXETINE	708	4.47 (4.14–4.82)	4.28 (1787.69)	4.25 (3.94)	2.09 (1.97)	<0.001	<0.001
	MONTELUKAST	690	7.05 (6.52–7.63)	6.54 (3257.21)	6.5 (6.01)	2.7 (2.57)	<0.001	<0.001
	BUPROPION	664	4.86 (4.49–5.26)	4.63 (1,899)	4.6 (4.25)	2.2 (2.08)	<0.001	<0.001
	SERTRALINE	659	3.23 (2.99–3.49)	3.14 (966.81)	3.12 (2.89)	1.64 (1.52)	<0.001	<0.001
	FAMPRIDINE	608	3.33 (3.07–3.61)	3.23 (941.22)	3.21 (2.96)	1.68 (1.56)	<0.001	<0.001
	ATOMOXETINE	565	4.14 (3.81–4.51)	3.98 (1269.86)	3.96 (3.64)	1.99 (1.85)	<0.001	<0.001
	NALTREXONE	547	3.85 (3.53–4.2)	3.72 (1092.8)	3.7 (3.39)	1.89 (1.75)	<0.001	<0.001
	LEDIPASVIR SOFOSBUVIR	534	3.55 (3.25–3.87)	3.43 (926.85)	3.42 (3.13)	1.77 (1.64)	<0.001	<0.001
	OMALIZUMAB	483	2.64 (2.41–2.89)	2.59 (473.37)	2.58 (2.35)	1.37 (1.23)	<0.001	<0.001
	DASABUVIR OMBITASVIR PARITAPREVIR RITONAVIR	471	7.83 (7.12–8.61)	7.2 (2531.53)	7.16 (6.51)	2.84 (2.68)	<0.001	<0.001
	VENLAFAXINE	451	3.16 (2.87–3.47)	3.07 (635.09)	3.06 (2.79)	1.61 (1.47)	<0.001	<0.001
	TASIMELTEON	446	17.99 (16.22–19.95)	14.77 (5768.53)	14.69 (13.25)	3.88 (3.68)	<0.001	<0.001
	BUPRENORPHINE NALOXONE	429	4.41 (4–4.86)	4.22 (1062.52)	4.2 (3.81)	2.07 (1.92)	<0.001	<0.001
	LEVOFLOXACIN	426	2.7 (2.45–2.98)	2.64 (438.46)	2.63 (2.39)	1.4 (1.25)	<0.001	<0.001
	LISDEXAMFETAMINE	424	4.78 (4.33–5.27)	4.56 (1186.42)	4.54 (4.11)	2.18 (2.03)	<0.001	<0.001
	CLONAZEPAM	397	4.55 (4.11–5.03)	4.35 (1032.22)	4.33 (3.91)	2.12 (1.95)	<0.001	<0.001
	LEVOTHYROXINE	391	5.99 (5.4–6.64)	5.63 (1499.94)	5.61 (5.06)	2.49 (2.32)	<0.001	<0.001
	SOFOSBUVIR VELPATASVIR	387	3.27 (2.95–3.62)	3.18 (582.66)	3.17 (2.86)	1.66 (1.51)	<0.001	<0.001
Female	LEVOTHYROXINE	3,865	6.71 (6.48–6.93)	6.16 (16,556)	6.03 (5.83)	2.59 (2.54)	<0.001	<0.001
	DULOXETINE	3,800	7.19 (6.95–7.43)	6.55 (17735.46)	6.42 (6.21)	2.68 (2.63)	<0.001	<0.001
	VARENICLINE	3,654	5.44 (5.26–5.63)	5.09 (11916.68)	4.99 (4.83)	2.32 (2.27)	<0.001	<0.001
	PREGABALIN	3,426	3.18 (3.07–3.29)	3.07 (4756.27)	3.03 (2.92)	1.6 (1.55)	<0.001	<0.001
	QUETIAPINE	2,977	4.66 (4.48–4.83)	4.4 (7807.54)	4.34 (4.18)	2.12 (2.06)	<0.001	<0.001
	FAMPRIDINE	2,175	3.5 (3.35–3.65)	3.36 (3618.93)	3.33 (3.19)	1.74 (1.67)	<0.001	<0.001
	NIRAPARIB	2048	12.21 (11.64–12.8)	10.37 (17402.71)	10.25 (9.78)	3.36 (3.28)	<0.001	<0.001
	SERTRALINE	1,527	3.32 (3.15–3.49)	3.2 (2,326)	3.18 (3.02)	1.67 (1.59)	<0.001	<0.001
	PAROXETINE	1,447	4.26 (4.04–4.49)	4.05 (3349.02)	4.02 (3.81)	2.01 (1.93)	<0.001	<0.001
	BUPROPION	1,400	3.99 (3.78–4.21)	3.81 (2923.99)	3.79 (3.59)	1.92 (1.84)	<0.001	<0.001
	VENLAFAXINE	1,327	3.24 (3.07–3.43)	3.13 (1941.16)	3.11 (2.95)	1.64 (1.56)	<0.001	<0.001
	ARIPIPRAZOLE	1,189	2.38 (2.24–2.52)	2.33 (907.66)	2.32 (2.19)	1.21 (1.13)	<0.001	<0.001
	LEVOFLOXACIN	1,126	4.09 (3.85–4.34)	3.9 (2445.3)	3.88 (3.65)	1.95 (1.86)	<0.001	<0.001
	ALENDRONIC ACID	1,116	2.5 (2.35–2.65)	2.44 (954.53)	2.43 (2.29)	1.28 (1.19)	<0.001	<0.001
	CIPROFLOXACIN	1,042	3.34 (3.14–3.55)	3.22 (1609.3)	3.2 (3.01)	1.68 (1.59)	<0.001	<0.001
	DESVENLAFAXINE	994	5.45 (5.11–5.81)	5.09 (3298.6)	5.06 (4.75)	2.34 (2.24)	<0.001	<0.001
	MONTELUKAST	886	5.12 (4.78–5.48)	4.81 (2698.68)	4.78 (4.47)	2.26 (2.15)	<0.001	<0.001
	CLONAZEPAM	883	5.57 (5.19–5.96)	5.19 (3017.83)	5.17 (4.82)	2.37 (2.26)	<0.001	<0.001
	PEGINTERFERON ALFA 2A	843	3.85 (3.59–4.13)	3.68 (1664.88)	3.67 (3.42)	1.88 (1.77)	<0.001	<0.001
	LORATADINE PSEUDOEPHEDRINE	671	10.64 (9.8–11.55)	9.23 (4981.86)	9.19 (8.47)	3.2 (3.06)	<0.001	<0.001
	LEUPRORELIN	653	2.44 (2.25–2.64)	2.38 (530.46)	2.38 (2.2)	1.25 (1.13)	<0.001	<0.001
	ALPRAZOLAM	638	2.3 (2.12–2.49)	2.25 (449.78)	2.25 (2.08)	1.17 (1.05)	<0.001	<0.001
	ANASTROZOLE	588	2.81 (2.59–3.05)	2.73 (652.68)	2.72 (2.51)	1.45 (1.32)	<0.001	<0.001
	ESCITALOPRAM	530	2.7 (2.48–2.95)	2.63 (542.24)	2.62 (2.41)	1.39 (1.26)	<0.001	<0.001
	CITALOPRAM	507	2.37 (2.17–2.59)	2.32 (384.37)	2.31 (2.12)	1.21 (1.08)	<0.001	<0.001
	LEFLUNOMIDE	468	2.74 (2.5–3.01)	2.67 (495.44)	2.67 (2.43)	1.41 (1.27)	<0.001	<0.001
	LEDIPASVIR SOFOSBUVIR	461	3.25 (2.96–3.57)	3.14 (680.21)	3.13 (2.85)	1.65 (1.5)	<0.001	<0.001
	FEXOFENADINE	448	2.59 (2.35–2.84)	2.52 (416.88)	2.52 (2.29)	1.33 (1.19)	<0.001	<0.001
	CLARITHROMYCIN	447	3.04 (2.77–3.35)	2.95 (582.42)	2.94 (2.67)	1.56 (1.41)	<0.001	<0.001
	ATOMOXETINE	406	4.56 (4.12–5.04)	4.32 (1048.07)	4.31 (3.89)	2.11 (1.95)	<0.001	<0.001

For the top 20 drugs ranked by the total number of insomnia reports across both sexes, we further evaluated sex–drug interaction using chi-square tests for equality of sex-specific log RORs (Wald test, df = 1) shown in Table 3. Significant sex differences were observed for 15 of the 20 drugs (*p* < 0.05). For most agents, including varenicline, eszopiclone, oxybate sodium, montelukast, duloxetine, pregabalin and quetiapine, the association with insomnia was stronger in males than in females (e.g., varenicline: ROR_m = 6.61 vs. ROR_f = 5.44; χ^2^ = 44.88, *p* < 0.0001). In contrast, levothyroxine and levofloxacin showed stronger signals in females (levothyroxine: ROR_m = 5.99 vs. ROR_f = 6.71; χ^2^ = 4.20, *p* = 0.0405). No statistically significant sex difference was detected for esomeprazole, fampridine, paroxetine, sertraline, or venlafaxine (all *p* ≥ 0.05).

**Table 3:** Sex–drug interaction analysis for the top 20 drugs associated with insomnia.

Drug	Male ROR (95% CI)	Female ROR (95% CI)	Chi-square (df = 1)	*p*-value	Interpretation
Varenicline	6.61 (6.32–6.92)	5.44 (5.26–5.63)	44.88	<0.0001	Higher in males
Eszopiclone	60.00 (56.09–64.18)	40.30 (38.34–42.35)	86.8	<0.0001	Markedly higher in males
Oxybate sodium	5.40 (5.00–5.83)	4.25 (4.09–4.41)	43.37	<0.0001	Higher in males
Levofloxacin	2.70 (2.47–2.95)	4.09 (3.85–4.34)	50.53	<0.0001	Higher in females
Montelukast	7.05 (6.44–7.73)	5.12 (4.78–5.48)	36.33	<0.0001	Higher in males
Aripiprazole	2.99 (2.77–3.23)	2.38 (2.24–2.52)	26.02	<0.0001	Higher in males
Peginterferon alfa-2a	5.12 (4.77–5.50)	3.85 (3.59–4.13)	34.29	<0.0001	Higher in males
Omalizumab	2.64 (2.46–2.84)	1.87 (1.76–1.98)	39.24	<0.0001	Higher in males
Gabapentin	2.12 (2.00–2.25)	1.72 (1.61–1.83)	15.03	0.0001	Higher in males
Bupropion	4.86 (4.54–5.20)	3.99 (3.78–4.21)	16.23	0.0001	Higher in males
Duloxetine	7.95 (7.50–8.43)	7.19 (6.95–7.43)	8.53	0.0035	Higher in males
Ciprofloxacin	3.80 (3.52–4.10)	3.34 (3.14–3.55)	6.72	0.0095	Slightly higher in males
Pregabalin	3.40 (3.23–3.59)	3.18 (3.07–3.29)	4.58	0.0324	Slightly higher in males
Quetiapine	4.97 (4.74–5.21)	4.66 (4.48–4.83)	4.38	0.0365	Slightly higher in males
Levothyroxine	5.99 (5.64–6.36)	6.71 (6.48–6.93)	4.2	0.0405	Slightly higher in females
Esomeprazole	1.63 (1.53–1.74)	1.50 (1.41–1.60)	1.99	0.1586	No significant sex difference
Fampridine	3.33 (3.14–3.53)	3.50 (3.35–3.65)	1.11	0.2926	No significant sex difference
Paroxetine	4.47 (4.23–4.73)	4.26 (4.04–4.49)	1.02	0.312	No significant sex difference
Sertraline	3.23 (3.05–3.41)	3.32 (3.15–3.49)	0.31	0.5766	No significant sex difference
Venlafaxine	3.16 (2.97–3.35)	3.24 (3.07–3.43)	0.23	0.6281	No significant sex difference

### 3.4 External validation with the Canadian database

**Figure 5:**
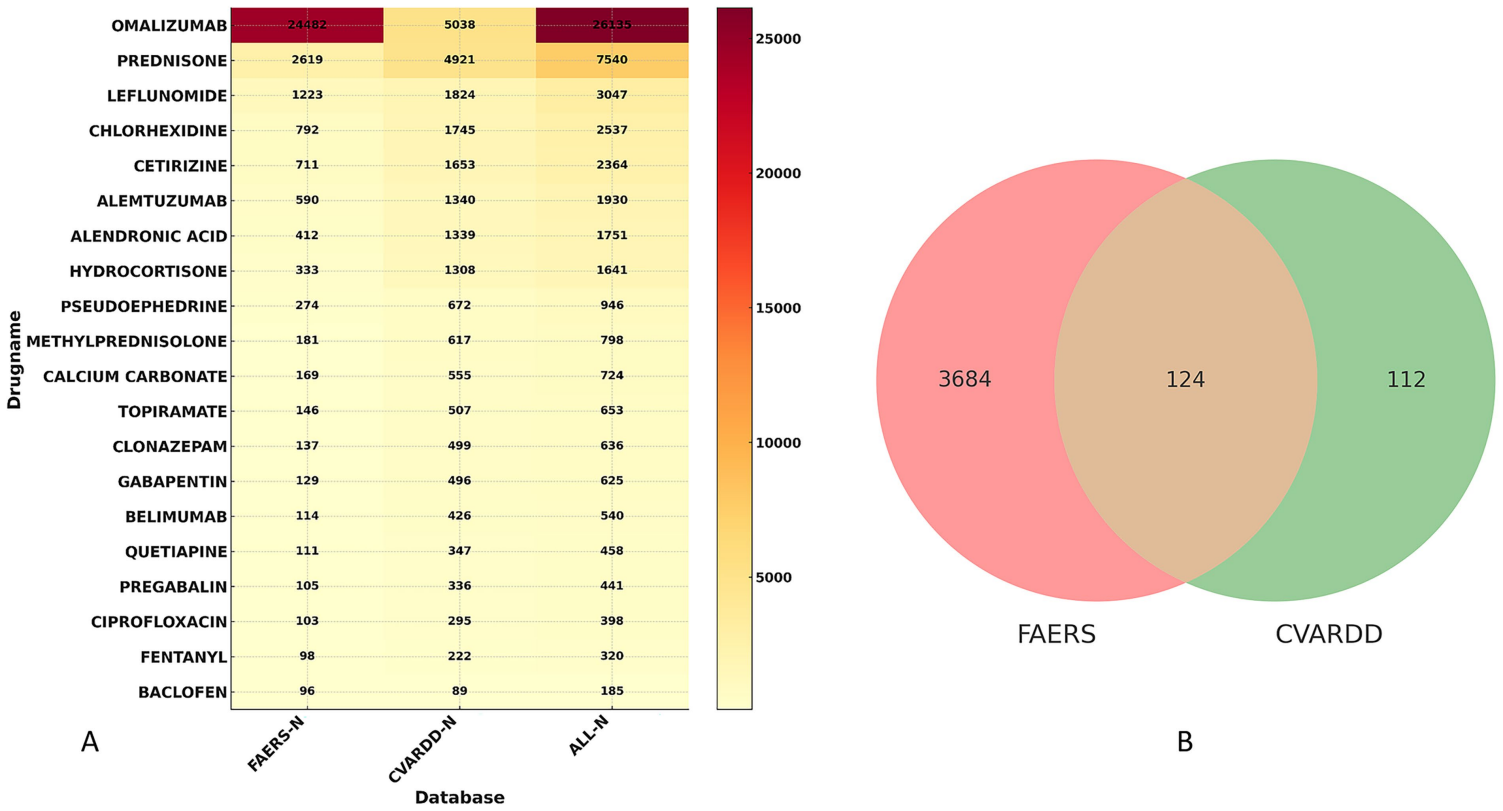
Insomnia Risk Drug Signals from FAERS and CVARDD. **(A)** Heatmap of insomnia risk drug signals from FAERS and CVARDD; **(B)** Venn diagram of insomnia risk drug signals from FAERS and CVARDD.

To further ensure the accuracy of our results, we analyzed insomnia adverse events in the Canadian database, CVARDD. As shown in Figure 5B, the FAERS database reported 3,808 drugs associated with insomnia, while the CVARDD database reported 236 drugs, with 124 drugs appearing in both databases. The data from both databases were merged to create the heatmap shown in Figure 5A. In the FAERS database, the drugs with the highest number of insomnia-related adverse events were OMALIZUMAB (*N* = 24,482), PREDNISONE (*N* = 2,619), LEFLUNOMIDE (*N* = 1,223), CHLORHEXIDINE (*N* = 792), and CETIRIZINE (*N* = 711). In the CVARDD database, the drugs with the highest number of insomnia-related adverse events were OMALIZUMAB (*N* = 5,038), PREDNISONE (*N* = 4,921), LEFLUNOMIDE (*N* = 1,824), CHLORHEXIDINE (*N* = 1,745), and CETIRIZINE (*N* = 1,653).

### 3.5 Time-to-onset analysis

**Figure 6:**
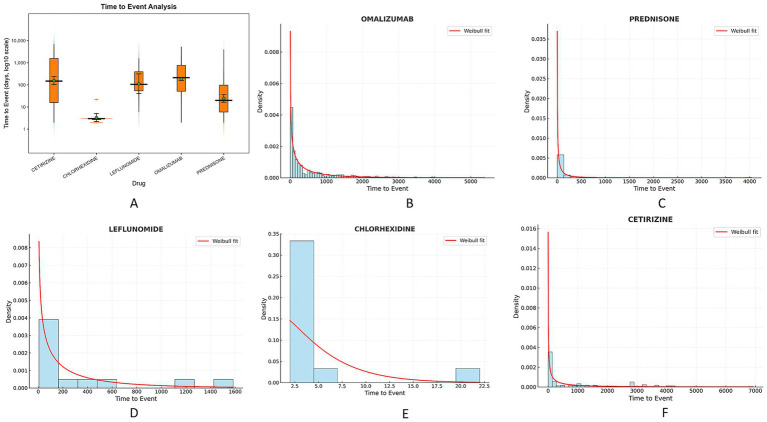
Analysis of drug induced time for high risk signals of insomnia. **(A)** Violin plot of induction time for 5 high-risk insomnia drugs; **(B)** Weibull distribution curve of the induction time of OMALIZUMAB insomnia risk drugs; **(C)** Prednisone insomnia risk drug induction time Weibull distribution curve; **(D)** The Weibull distribution curve of LEFLUNOMIDE insomnia risk drug induction time; **(E)** CHLORHEXIDINE Insomnia Risk Drug Induction Time Weibull Distribution Curve; **(F)** Weibull distribution curve of CETIRIZINE insomnia risk drug induction time.

Time-to-onset analysis of adverse drug reactions is of significant importance for drug safety monitoring, clinical drug guidance, regulatory decision-making, and drug development improvements. We focused on the top five drugs in FAERS related to insomnia: OMALIZUMAB, PREDNISONE, LEFLUNOMIDE, CHLORHEXIDINE, and CETIRIZINE. As shown in Table 4, the shape parameters (β) for all drugs were less than 1, indicating that they exhibited early failure characteristics, with the risk primarily concentrated in the early stages of drug use. Among these, OMALIZUMAB had the most reports (*N* = 24,482), with a median time-to-onset of 211.5 days, while CHLORHEXIDINE had a median time-to-onset of 3 days, and PREDNISONE had a median of 20 days. We created a violin plot shown in Figure 6A, which indicates outliers for OMALIZUMAB and CETIRIZINE. Considering individual differences within the population, the Weibull distribution curves shown in Figures 6B–F all demonstrate early failure patterns.

**Table 4:** Weibull distribution of DII induction time.

Drug	TTO(days)		Weibull distribution		
	Case reports	Median (day)	Scale parameter: α (95%CI)	Shape parameter: β (95%CI)	Type
OMALIZUMAB	24,482	211.5	430.41 (372.04 ~ 488.78)	0.645 (0.61 ~ 0.70)	Early failure
PREDNISONE	2,619	20	65.27 (35.72 ~ 94.83)	0.49 (0.42 ~ 0.56)	Early failure
LEFLUNOMIDE	1,223	106	254.13 (35.89 ~ 472.27)	0.67 (0.39 ~ 0.94)	Early failure
CHLORHEXIDINE	792	3	219.72 (107.21 ~ 332.22)	0.47 (0.41 ~ 0.54)	Early failure
CETIRIZINE	711	148	525.34 (324.50 ~ 726.18)	0.74 (0.51 ~ 0.97)	Early failure

TTO, time to onset; CI, confidence interval.

## 4 Discussion

Insomnia is a highly prevalent sleep disorder associated with substantial physical and psychological morbidity, including depression, anxiety, cardiovascular disease, metabolic disturbances, and increased all-cause mortality (22). Numerous medications can trigger or exacerbate insomnia through diverse neurobiological pathways. Central nervous system stimulants alter dopamine and monoamine balance (23); glucocorticoids and endocrine-modulating agents disrupt hypothalamic–pituitary–adrenal and thyroid rhythms (8); and certain immunosuppressants or anticancer agents may influence sleep regulation via inflammatory or metabolic pathways (24). Using FAERS data from 2004Q1–2025Q2, this study provides the largest sex-stratified pharmacovigilance evaluation of DII to date, analyzing reporting trends, drug-specific signals, sex differences, and time-to-onset patterns based on 266,429 reports.

### 4.1 Susceptible populations and overall burden

The demographic patterns observed in FAERS highlight clear population-level disparities in susceptibility to drug-induced insomnia. Women accounted for a substantially higher proportion of DII reports, consistent with epidemiological evidence showing that females experience greater vulnerability to sleep disturbances due to hormonal fluctuations, stress reactivity, and differential drug metabolism (25). Adults aged 18–65 years represented the largest age group affected, likely reflecting higher exposure to long-term pharmacotherapy and psychosocial stressors, while older adults also constituted an important at-risk population given polypharmacy and age-related changes in sleep regulation. Although body weight distribution suggested a modest concentration among individuals ≥75 kg, extensive missing data limit the interpretability of weight-related patterns.

Patterns in reported indications further underscore the role of chronic inflammatory and autoimmune conditions—such as multiple sclerosis and rheumatoid arthritis—in predisposing patients to insomnia. These populations frequently receive long-term immunomodulatory or neuroactive therapies, heightening the risk for treatment-related sleep disturbances (26).

At the individual drug level, several widely used agents, including methotrexate, dupilumab, ciprofloxacin, teriparatide, and levofloxacin, appeared prominently among DII reports. Prior literature demonstrates that methotrexate may exert bidirectional effects on sleep: while reductions in systemic inflammation may improve sleep quality in rheumatoid arthritis (27), chemotherapy regimens containing methotrexate have been associated with worsened sleep and increased fatigue (28). These divergent findings suggest that drug-related sleep effects are context-dependent and influenced by underlying disease state, treatment intensity, and individual physiological variability.

Analysis by ATC classification revealed that central nervous system drugs accounted for the greatest proportion of insomnia reports, followed by antineoplastic and immunomodulating agents. This distribution aligns with clinical observations that neuroactive medications and treatments for chronic inflammatory or oncologic conditions frequently interfere with sleep–wake regulation (7, 29). Collectively, these patterns highlight the need for drug class–specific risk assessment that considers both pharmacological mechanisms and disease-specific factors contributing to insomnia vulnerability.

### 4.2 Gender differences and potential mechanisms

Clear sex-specific patterns emerged across drug–insomnia associations. In males, duloxetine showed one of the strongest insomnia signals, consistent with case reports describing duloxetine-induced disturbances in REM sleep regulation, including reversible REM sleep behavior disorder, likely mediated through serotonergic and noradrenergic pathways (30). Other drugs with prominent male-specific signals included montelukast, varenicline, and several neuropsychiatric agents, suggesting that men may be particularly sensitive to insomnia triggered by central stimulants, smoking-cessation therapies, and selected immunomodulators. In contrast, medications such as enzalutamide and carbidopa/levodopa exhibited more modest effects in males.

In females, antidepressants and endocrine-related therapies produced the most pronounced signals. Duloxetine also ranked among the highest-risk drugs, and levothyroxine demonstrated a strong association, potentially reflecting vulnerability linked to interactions between thyroid hormones and sleep regulation. Notably, niraparib showed a marked female-specific signal and was largely absent in males, aligning with reports of frequent insomnia during ovarian cancer treatment (31). Elevated signals for desvenlafaxine, paroxetine, and bupropion further indicate that serotonergic and noradrenergic modulation may disproportionately affect women.

Drugs such as quetiapine, duloxetine, and pregabalin showed consistent signals across multiple algorithms in both sexes, but with generally higher effect sizes in females. These sex differences may reflect variations in pharmacokinetics, including slower gastric emptying, higher body fat percentage, and differences in CYP enzyme expression, as well as hormonal influences on neurotransmitter systems (13). Overall, the patterns suggest that men may exhibit heightened sensitivity to insomnia associated with stimulants, smoking-cessation agents, and immunomodulators, whereas women appear more susceptible to insomnia triggered by antidepressants, endocrine therapies, and anticancer agents.

### 4.3 Potential risk drugs and clinical implications

Several widely used medications not currently labeled for insomnia-related adverse effects—such as pregabalin, quetiapine, gabapentin, omalizumab, and esomeprazole—showed notable disproportionality signals in FAERS. Their widespread use across neurological, psychiatric, immunologic, and gastrointestinal indications suggests that insomnia associated with these agents may be underrecognized in routine clinical settings.

The potential mechanisms underlying these associations vary across drug classes. Although pregabalin and gabapentin reduce neuronal excitability via modulation of voltage-gated calcium channels, they may paradoxically alter arousal thresholds or sleep architecture in susceptible individuals (32). Quetiapine, despite its sedative properties, has been linked to paradoxical insomnia through complex dopaminergic and serotonergic interactions. Omalizumab may influence sleep indirectly by modifying inflammatory or immunologic pathways, while long-term proton pump inhibitor use, such as esomeprazole, may affect micronutrient balance or endocrine function, potentially disrupting sleep homeostasis (33).

These findings have important clinical implications. For patients receiving long-term therapy with these agents—especially those with a history of sleep disturbances—clinicians should monitor changes in sleep patterns and counsel patients about potential insomnia risks. Treatment adjustments may be warranted when sleep disruption affects quality of life or therapeutic adherence. From a regulatory perspective, the identification of strong but unlabeled signals underscores the need for enhanced post-marketing surveillance, targeted pharmacoepidemiologic investigations, and consideration of updating drug labels should these associations be confirmed in independent data sources.

### 4.4 Time-to-onset analysis and risk management

The time-to-onset analysis demonstrated that several frequently reported drugs—including omalizumab, prednisone, leflunomide, chlorhexidine, and cetirizine—followed an early-failure pattern, indicating that insomnia generally emerges shortly after treatment initiation. Although the timing varied across agents, the concentration of early cases suggests that sleep disturbances may arise as an acute response to pharmacologic or immunologic perturbations rather than as cumulative long-term effects.

Omalizumab showed a particularly distinctive temporal profile. Given the intricate interactions between the immune and nervous systems (22)—such as mast cell–mediated neuropeptide signaling—omalizumab may influence sleep through immune–neural pathways, potentially contributing to early-phase insomnia in susceptible individuals (34). Prednisone and chlorhexidine also demonstrated rapid onset patterns, consistent with known mechanisms by which corticosteroids and inflammatory modulators can alter endocrine balance or provoke neuroimmune activation soon after exposure. Leflunomide and cetirizine exhibited moderately delayed but still early-phase clustering, reinforcing the overall early-failure tendency.

From a clinical standpoint, these temporal characteristics highlight the need for proactive early monitoring of sleep disturbances when initiating therapies associated with early-onset risks. Patients with pre-existing sleep disorders, psychiatric comorbidities, or immune dysregulation may warrant closer observation and anticipatory guidance. Early identification allows timely dose adjustments, supportive interventions, or alternative therapeutic strategies. More broadly, these TTO profiles offer valuable information for refining pharmacovigilance practices, enhancing patient counseling, and guiding risk-mitigation efforts in clinical settings.

### 4.5 Limitations

This study has several important limitations that should be considered when interpreting the findings. First, both FAERS and CVARDD are spontaneous reporting systems and are subject to well-known biases, including underreporting, selective reporting, stimulated reporting, and differential reporting behavior across sexes. These factors affect the completeness, accuracy, and comparability of the data. In addition, essential clinical information—such as dosage, treatment duration, temporal sequence of drug administration, lifestyle habits, comorbid sleep disorders, and psychiatric history—is frequently missing, limiting the ability to contextualize insomnia onset or to adjust for individual-level confounders.

Second, the analyses could not account for confounding by indication, which is particularly relevant because many high-signal drugs (e.g., antidepressants, anticancer agents, immunomodulators) are prescribed for conditions inherently associated with insomnia. Without access to disease severity, baseline insomnia status, or alternative treatment options, it is not possible to determine whether the observed signals reflect drug effects, underlying disease, or their interaction.

Moreover, the FAERS database does not provide denominator data, such as the number of exposed patients stratified by sex or by individual drug. Therefore, incidence rates and absolute risks cannot be estimated, and disproportionality measures should be interpreted as signal-detection metrics rather than estimates of causal effect. Multireported events, concomitant medications, and polypharmacy—common in psychiatric and chronic disease populations—could not be disentangled or systematically controlled. Although this study restricted analyses to “Primary Suspect” drugs to reduce noise, this approach may underrepresent multi-drug contributions to insomnia.

From a methodological perspective, disproportionality analysis inherently provides hypothesis-generating results rather than causal inference. Sex differences derived from ROR comparisons may also be influenced by unequal reporting volumes, especially given the higher proportion of female reports, potentially inflating apparent sex differences. Finally, the external validation in CVARDD used the same disproportionality framework, which strengthens consistency but does not eliminate confounding or verify causality. Prospective pharmacoepidemiologic studies are needed to confirm these signals in real-world clinical populations.

## 5 Conclusion

This study used large-scale spontaneous reporting data from FAERS and CVARDD to identify drug–insomnia associations, evaluate sex-specific differences, validate signals across independent sources, and describe time-to-onset patterns for frequently reported drugs. Several commonly used medications demonstrated notable or sex-specific disproportionality signals, including agents not currently labeled for insomnia-related adverse effects. Time-to-onset analyses indicated that insomnia generally arises early in treatment for multiple drugs.

Given the inherent limitations of spontaneous reporting systems, these findings should be interpreted as signal-generating rather than confirmatory. Further epidemiologic and mechanistic studies are warranted to validate these associations and to inform the development of sex-sensitive approaches to medication safety.

## Data Availability

The original contributions presented in the study are included in the article/Supplementary material, further inquiries can be directed to the corresponding author.

## References

[1] Brewster GS Riegel B Gehrman PR. Insomnia in the older adult. Sleep Med Clin. (2022) 17:233–9. doi: 10.1016/j.jsmc.2022.03.004, 35659076

[2] Palagini L Arango C Bassetti CLA Bastien C Geoffroy PA Elder G . The need to prioritize "insomnia disorder" in public health agendas: "a wakeup call" position paper from European and Canadian experts in sleep and mental health. Sleep Med. (2025) 135:106763. doi: 10.1016/j.sleep.2025.106763, 40886655

[3] Benjafield AV Sert Kuniyoshi FH Malhotra A Martin JL Morin CM Maurer LF . Estimation of the global prevalence and burden of insomnia: a systematic literature review-based analysis. Sleep Med Rev. (2025) 82:102121. doi: 10.1016/j.smrv.2025.102121, 40627924 PMC12676268

[4] Morin CM Jarrin DC. Epidemiology of insomnia: prevalence, course, risk factors, and public health burden. Sleep Med Clin. (2022) 17:173–91. doi: 10.1016/j.jsmc.2022.03.003, 35659072

[5] Van Gastel A. Drug-induced insomnia and excessive sleepiness. Sleep Med Clin. (2022) 17:471–84. doi: 10.1016/j.jsmc.2022.06.011, 36150808

[6] Foral P Knezevich J Dewan N Malesker M. Medication-induced sleep disturbances. Consult Pharm. (2011) 26:414–25. doi: 10.4140/TCP.n.2011.414, 21628140

[7] Zhou S Li P Lv X Lai X Liu Z Zhou J . Adverse effects of 21 antidepressants on sleep during acute-phase treatment in major depressive disorder: a systemic review and dose-effect network meta-analysis. Sleep. (2023) 46:10.1093/sleep/zsad177. doi: 10.1093/sleep/zsad177, 37422714 PMC10566234

[8] Szmyd B Rogut M Białasiewicz P Gabryelska A. The impact of glucocorticoids and statins on sleep quality. Sleep Med Rev. (2021) 55:101380. doi: 10.1016/j.smrv.2020.101380, 33010620

[9] Ramin T Peter J Schneider M Dahling V Zolk O. Age and sex differences in adverse events associated with antipsychotics: an analysis of the FDA adverse events database. Int J Geriatr Psychiatry. (2025) 40:e70142. doi: 10.1002/gps.70142, 40817421 PMC12356759

[10] Beery AK Zucker I. Sex bias in neuroscience and biomedical research. Neurosci Biobehav Rev. (2011) 35:565–72. doi: 10.1016/j.neubiorev.2010.07.002, 20620164 PMC3008499

[11] Waxman DJ Holloway MG. Sex differences in the expression of hepatic drug metabolizing enzymes. Mol Pharmacol. (2009) 76:215–28. doi: 10.1124/mol.109.056705, 19483103 PMC2713118

[12] Baker FC Lee KA. Menstrual cycle effects on sleep. Sleep Med Clin. (2022) 17:283–94. doi: 10.1016/j.jsmc.2022.02.004, 35659080

[13] Harrington YA Parisi JM Duan D Rojo-Wissar DM Holingue C Spira AP. Sex hormones, sleep, and memory: interrelationships across the adult female lifespan. Front Aging Neurosci. (2022) 14:800278. doi: 10.3389/fnagi.2022.800278, 35912083 PMC9331168

[14] Pajėdienė E Urbonavičiūtė V Ramanauskaitė V Strazdauskas L Stefani A. Sex differences in insomnia and circadian rhythm disorders: a systematic review. Medicina (Kaunas). (2024) 60:10.3390/medicina60030474. doi: 10.3390/medicina60030474, 38541200 PMC10971860

[15] Marver JE Mcglinchey EA. Sex differences in insomnia and risk for psychopathology in adolescence. Curr Opin Psychol. (2020) 34:63–7. doi: 10.1016/j.copsyc.2019.09.004, 31655365

[16] Thiruchelvam T Lim CX Munro C Chan V Jayasuria G Coulthard KP . Adverse events and drug interactions associated with Elexacaftor/Tezacaftor/ivacaftor treatment: a descriptive study across Australian, Canadian, and American adverse event databases. Life (Basel). (2025) 15:10.3390/life15081256. doi: 10.3390/life15081256, 40868904 PMC12387162

[17] Rothman KJ Lanes S Sacks ST. The reporting odds ratio and its advantages over the proportional reporting ratio. Pharmacoepidemiol Drug Saf. (2004) 13:519–23. doi: 10.1002/pds.1001, 15317031

[18] Evans SJ Waller PC Davis S. Use of proportional reporting ratios (PRRs) for signal generation from spontaneous adverse drug reaction reports. Pharmacoepidemiol Drug Saf. (2001) 10:483–6. doi: 10.1002/pds.677, 11828828

[19] Bate A Lindquist M Edwards IR Olsson S Orre R Lansner A . A Bayesian neural network method for adverse drug reaction signal generation. Eur J Clin Pharmacol. (1998) 54:315–21. doi: 10.1007/s002280050466, 9696956

[20] Lin Z Xue J Yang M Yu X Zhong J. Safety assessment of laronidase: real-world adverse event analysis based on the FDA adverse event reporting system (FAERS). Front Pharmacol. (2025) 16:1623921. doi: 10.3389/fphar.2025.1623921, 40910004 PMC12405411

[21] Yang H Huang R Zhang P Liu Y Liu Z He J . Association between statin use and immune-related adverse events in patients treated with immune checkpoint inhibitors: analysis of the FAERS database. Front Immunol. (2024) 15:1439231. doi: 10.3389/fimmu.2024.1439231, 39439792 PMC11493589

[22] Tang N Zeng Y He G Chen S. Interference between immune cells and insomnia: a bibliometric analysis from 2000 to 2023. Front Neurol. (2025) 16:1486548. doi: 10.3389/fneur.2025.1486548, 40206297 PMC11978667

[23] Ye X Pang S Ren X Wang H Chen M. Neurotransmitter modulation of sleep-wake states: from molecular mechanisms to therapeutic potential. Sleep Med. (2025) 132:106547. doi: 10.1016/j.sleep.2025.106547, 40359849

[24] Rohde KA Schlei ZW Katers KM Weber AK Brokhof MM Hawes DS . Insomnia and relationship with immunosuppressant therapy after lung transplantation. Prog Transplant. (2017) 27:167–74. doi: 10.1177/1526924817699960, 28617161

[25] Grandner MA. Sleep, health, and society. Sleep Med Clin. (2017) 12:1–22. doi: 10.1016/j.jsmc.2016.10.012, 28159089 PMC6203594

[26] Drerup M Roth A Kane A Sullivan AB. Therapeutic approaches to insomnia and fatigue in patients with multiple sclerosis. Nat Sci Sleep. (2021) 13:201–7. doi: 10.2147/NSS.S256676, 33623461 PMC7896778

[27] Straub RH Detert J Dziurla R Fietze I Loeschmann PA Burmester GR . Inflammation is an important covariate for the crosstalk of sleep and the HPA Axis in rheumatoid arthritis. Neuroimmunomodulation. (2017) 24:11–20. doi: 10.1159/000475714, 28535535

[28] Kuo H Chiu M Liao W Kuo H-H Chiu M-J Liao W-C . Quality of sleep and related factors during chemotherapy in patients with stage I/II breast cancer. J Formos Med Assoc. (2006) 105:64–9. doi: 10.1016/S0929-6646(09)60110-8, 16440072

[29] Singh KK Ghosh S Bhola A Verma P Amist AD Sharma H . Sleep and immune system crosstalk: implications for inflammatory homeostasis and disease pathogenesis. Ann Neurosci. (2025) 32:196–206. doi: 10.1177/09727531241275347, 39544655 PMC11559494

[30] Tan L Zhou J Yang L Ren R Zhang Y Li T . Duloxetine-induced rapid eye movement sleep behavior disorder: a case report. BMC Psychiatry. (2017) 17:372. doi: 10.1186/s12888-017-1535-4, 29162053 PMC5698922

[31] Monk BJ González-Martin A Buckley L Matulonis UA Rimel BJ Wu X . Safety and management of niraparib monotherapy in ovarian cancer clinical trials. Int J Gynecol Cancer. (2023) 33:971–81. doi: 10.1136/ijgc-2022-004079, 36792166 PMC10313963

[32] Wang T Yin D Guo W Wang TX Liu YY Li YD . Antinociceptive and hypnotic activities of pregabalin in a neuropathic pain-like model in mice. Pharmacol Biochem Behav. (2015) 135:31–9. doi: 10.1016/j.pbb.2015.05.007, 25989046

[33] Smaoui H Chtourou L Jallouli D Jemaa SB Karaa I Boudabbous M . Effect of long-term proton pump inhibitors on phosphocalcium metabolism and bone mineral density. Future Sci OA. (2024) 10:FSO977. doi: 10.2144/fsoa-2023-0198, 38841182 PMC11152587

[34] Yavuz GO Yılgör A Yavuz IH Milanlıoğlu A Çilingir V Çağaç A . Effects of omalizumab therapy on peripheral nerve functions: short observational study. Postepy Dermatol Alergol. (2019) 36:211–6. doi: 10.5114/ada.2018.74834, 31320856 PMC6627259

